# Informal caregiving and personality: Results of a population-based longitudinal study in Germany

**DOI:** 10.1371/journal.pone.0203586

**Published:** 2018-09-06

**Authors:** André Hajek, Hans-Helmut König

**Affiliations:** Department of Health Economics and Health Services Research, Hamburg Center for Health Economics, University Medical Center Hamburg-Eppendorf, Hamburg, Germany; University of Edinburgh, UNITED KINGDOM

## Abstract

**Background:**

The aim of this study was to identify whether informal caregiving time is associated with personality factors longitudinally.

**Methods:**

Longitudinal data were gathered from the German Socio-Economic Panel (GSOEP), a large nationally representative, longitudinal study of German households beginning in 1984. Focusing on the association between informal caregiving and personality factors, data were used from the years 2005, 2009 and 2013. The GSOEP Big Five Inventory was used to assess personality factors. Informal caregiving hours were used as explanatory variable. The explanatory variable informal caregiving hours was categorized into 0 hours (reference), 1 hours, 2 hours, 3 hours, 4 hours, and 5 hours and more. Age, marital status, educational level, employment status, income, self-rated health and disability were included as potential confounders in regression analysis.

**Results:**

Adjusting for potential confounders, fixed effects regressions showed that whether or not someone provides informal care is markedly associated with changes in neuroticism. Given that an individual provides informal care, the actual number of care hours did not matter in most cases. Informal caregiving was not associated with openness to experience, extraversion and agreeableness. As regards conscientiousness, only ‘5 hours and more’ on a typical Sunday was associated with an increase in conscientiousness (β = .32, p < .05). Informal caregiving on a typical weekday or Saturday was not associated with changes in conscientiousness.

**Conclusion:**

Our findings stress the longitudinal association between informal caregiving and neuroticism.

## Introduction

Given that there is a need for care, the majority of individuals would like to be cared for at home [1, 2]. In Germany, care at home is mostly provided by informal caregivers [3, 4]. With demographic changes of the aging population, the need for long-term care is expected to rise very rapidly, emphasizing the meaning of informal caregiving. Starting informal care, however, is associated with various adverse health outcomes [5–9].

While the consequences of informal caregiving on health outcomes have been studied extensively thus far, little is known about the consequences on personality factors. It is widely accepted that *Agreeableness*, *Conscientiousness*, *Extraversion*, *Neuroticism* and *Openness to Experience* can be considered as main personality factors [10, 11]. Openness to experience refers to the tendency to be imaginative, and open-minded. Neuroticism refers to the tendency to experience negative emotions (e.g., depressive symptoms or anger). Extraversion refers to the tendency to be energetic, assertive, gregarious and outgoing. Conscientiousness refers to the tendency to be organized, prepared, responsible and persistent. Agreeableness refers to the tendency to be forgiving, kind, trusting and cooperative. There is some evidence [12, 13] showing that personality can change over time. Thus, personality factors can be considered as factors varying within individuals over time that are modifiable.

In our view, it is worth studying the relation between informal caregiving and personality factors for two main reasons. First, the literature on informal caregiving mainly focuses on health-related outcomes. We argue that it is worth investigating the factors associated with changes in informal caregiving in a broader sense. Second, there was a tendency to regard the personality factors as relatively fixed. However, a recent study has shown that changes in unemployment are associated with changes in personality factors [12]. More generally, studies aim to identify life circumstances and life events associated with changes in personality factors [14]. We argue that it is worth studying the factors that are associated with changes in personality factors. For example, changes in personality factors are associated with changes in health outcomes including cognitive health and mortality [15] and health care burden [16].

While several studies have examined the influence of caregiver personality on the burden of family caregivers [17, 18], little is known about whether informal care is associated with the personality of the caregiver over time [19]. Based on two waves of the GSOEP (2005 and 2009), one recent study has found that individuals scoring high in neuroticism are more likely to take over the responsibility to provide informal care [19]. However, little is known about the relation between informal caregiving time and personality factors longitudinally. Longitudinal studies are required to examine changes within individuals over time. Moreover, the problem of unobserved heterogeneity which is a main problem in cross-sectional studies can be reduced when panel regression models are used [20]. Hence, based on a large nationally representative sample, the aim of the present study was to investigate whether informal caregiving time were associated with personality factors longitudinally. Knowledge about a relation between informal caregiving time and personality might underline the importance of replacing informal caregiving with, e.g., paid home care workers.

We hypothesize that increases in informal caregiving time are associated with an increase in neuroticism. This might be explained by the fact that increasing neuroticism is associated with increased negative emotions and increasing depressive symptoms. It has particularly been demonstrated that starting informal care is associated with an increase in depressive symptoms [5]. In addition, we hypothesize that increases in informal caregiving time are associated with an increase in conscientiousness. A possible explanation might be that increases in informal caregiving time require higher level of structuredness to manage multiple life domains (e.g., to provide informal care, to fulfill family obligations, to devote time to hobbies, or to participate in cultural life). Moreover, we hypothesize that increases in informal caregiving time are associated with a decrease in extraversion. This is conceivable because increases in caregiving time might markedly restrict leisure time. This restriction might lead to withdrawal or disinterest. Moreover, the caregivers might become less outgoing. However, it also appears plausible that increasing caregiving time leads to an increased desire to escape tasks. Thus, this hypothesis is explorative. The relation between informal caregiving time and openness to experience and agreeableness were investigated in an explorative manner.

## Methods

### Sample

For the current study, data were retrieved from the German Socio-Economic Panel (GSOEP), located at the German Institute for Economic Research, DIW Berlin. It is a household panel like the British Household Panel Study (BHPS) or the PSID (Panel Study of Income Dynamics in the US). Starting in 1984, almost 11,000 households and over 20,000 individuals are being interviewed annually, covering Germans living in the Old and New German States, foreigners, and recent immigrants to Germany. In the GSOEP, all adult household members (aged 17 and over) are interviewed. Topics are, for example, social exclusion, domain satisfaction, occupational status, and health. Response rates are very high [21] and survey attrition is low for the GSOEP [22]. Our analysis is restricted to 2005, 2009 and 2013 as personality factors were quantified only in these waves. Concerning the sampling frame and survey design further details are provided elsewhere [23]. This survey is approved as being in accordance with the standards of the Federal Republic of Germany for lawful data protection. Participants gave free and informed consent to participate in the survey.

An ethical approval was not obtained because criteria for the need of an ethical statement were not met (risk for the respondents, lack of information about the aims of the study, examination of patients). However, the German Council of Science and Humanities (Wissenschaftsrat) evaluated the German Socio-Economic Panel (GSOEP) at the Deutsches Institut für Wirtschaftsforschung, (DIW), Berlin. The German Council of Science and Humanities approved the GSOEP.

### Dependent variables

The 15-item short version of the Big Five Inventory (BFI-S) [24] was used to measure personality (openness, conscientiousness, extraversion, agreeableness, and neuroticism) with three items per dimension in the current study. The statements were rated on a 7-point scale ranging from do not at all agree to fully agree. Each dimension is measured by the sum of three items (ranging from 3 to 21). The BFI-S is based on the 44-item Big Five Inventory [25]. It has been demonstrated that the psychometric properties of the BFI-S were acceptable [26].

### Independent variables

Informal caregiving hours were quantified using the question: “What is a typical day like for you? How many hours do you spend on the following activities on a typical weekday, Saturday, and Sunday?”. Apart from other activities, individuals reported the number of hours for “care and support for persons in need of care” (a) on a typical weekday, (b) Saturday, and (c) Sunday, each ranging from 0 to 24 hours. The explanatory variable informal caregiving hours was categorized into 0 hours (reference), 1 hours, 2 hours, 3 hours, 4 hours, and 5 hours and more.

Age, gender, marital status (Married, living together with my spouse; others (Married, living separated from spouse; single; divorced; widowed), employment status (Ref.: unemployed) and educational level (ISCED-97 (International Standard Classification of Education), which ranges from 0 to 6; with low (0–2; respondents without formal vocational qualification), medium (3–4; respondents with vocational training (at work/at school), including respondents with a higher general school certificate without professional training) and high (5–6; individuals with completed professional development training [professional, master or technical school, university of cooperative education, or academies] and respondents with completed university studies [university or university of applied science]) education) [27] were used. The (log) square root equivalence scale (total household net income is divided by the square root of household size) was used to quantify income.

Self-rated health was measured using a 5-point Likert scale which ranges from 1 = “very good” to 5 = “bad”. It was treated as ordinal variable. The question “Are you legally classified as handicapped or capable of gainful employment only to a reduced extent due to medical reasons? (yes; no) was used to assess disability. It was considered as a surrogate for morbidity [28–30] because physician diagnosed illnesses were only assessed in the years 2009, 2011 and 2013.

### Statistical analysis

Unobserved heterogeneity is a fundamental problem in observational studies, in particular in those relying on cross-sectional data. For example, genetic disposition might bias cross-sectional studies based on large surveys because this factor is almost impossible to assess in large survey studies [20]. However, panel regression models exist dealing with unobserved factors. The fixed effects (FE) estimator provides consistent estimates if there is unobserved heterogeneity that is correlated with the variable of interest, but which is constant within individuals [31]. This is a main reason why FE regressions are popular and widely used among, e.g., microeconometricians or sociologists. We also used FE regressions in this study. This choice was supported by Sargan-Hansen tests (for example, with neuroticism as outcome measure, Sargan-Hansen statistic was 778.8, p < .001). The Sargan-Hansen test is a Hausman test [32] additionally allowing for cluster-robust standard errors.

Within the FE framework, exclusively changes within units or individuals over time were used. Between-variation is wiped out and consequently not used for FE model estimates. Therefore, only factors varying within individuals over time can be included as main effects.

It is worth noting that time-varying unobserved heterogeneity can bias the estimates. This is not just a hypothetical problem, but is a recognized challenge. For example, anticipation effects or emotional effects can bias the FE estimates [33–35]. For example, when individuals anticipate losing their job, they might shift their time from paid work to informal care obligations. Simultaneously, they might not suffer emotionally due to informal caregiving, but since they have to adapt their career goals and aspirations.

The level of significance was set at α = .05. Statistical analysis was performed using Stata Release 14 (Stata Corp., College Station, Texas).

## Results

### Sample characteristics

Pooled sample characteristics for individuals included in FE regression analysis are shown in Table 1 (55,047 observations).

**Table 1:** Sample characteristics for individuals included in fixed effects regressions (2005, 2009 and 2013, pooled; 55,047 observations).

Variables	N (%) / Mean (SD)
Female: N (%)	28,916 (52.5%)
Age (in years): Mean (SD)	50.1 (17.6)
Married, living together with spouse: N (%)	22,133 (40.2)
Unemployed: N (%)	3,407 (6.2)
Low education: N (%)	9,526 (17.3)
Medium education: N (%)	29,573 (53.7)
High education: N (%)	15,948 (29.0)
Equivalence income: Mean (SD)	2,084.2 (1,718.3)
Self-rated health (from 1 = “very good” to 5 = “very bad”): Mean (SD)	2.7 (1.0)
Not severely disabled: N (%)	47,902 (87.0)
Neuroticism (higher values indicate higher neuroticism): Mean (SD)	11.6 (3.7)
Extraversion (higher values indicate higher extraversion): Mean (SD)	14.5 (3.4)
Openness to experience (higher values indicate higher openness): Mean (SD)	13.5 (3.6)
Agreeableness (higher values indicate higher agreeableness): Mean (SD)	16.2 (2.9)
Conscientiousness (higher values indicate higher conscientiousness): Mean (SD)	17.6 (2.8)
Informal care (hours on a typical weekday): Mean (SD); Range	0.2 (1.0); 0–24
Informal care (hours on a typical Saturday): Mean (SD); Range	0.2 (1.1); 0–24
Informal care (hours on a typical Sunday): Mean (SD); Range	0.2 (1.1); 0–24

Comments: The explanatory variable sex was not included in FE regressions as independent variable as it is time-constant (i.e., it usually did not vary within individuals over time). It was only used for descriptive purposes.

In total, slightly more than one-half were female (52.5%) and had a medium education (53.7%). Average age was 50.1 years (±17.6 years), ranging from 17 to 103 years. About 40% of the observations were married, living together with spouse. Average neuroticism score was 11.6 (±3.7), average extraversion score was 14.5 (±3.4), average openness to experience score was 13.5 (±3.6), average agreeableness score was 16.2 (±2.9), and average conscientiousness score was 17.6 (±2.8). Informal caregiving hours on a typical weekday equaled 0.2 (±1.0), and informal caregiving hours on a typical Saturday and Sunday was 0.2 (±1.1), ranging from 0 to 24 hours. Further details are shown in Table 1.

Moreover, it is worth noting that informal care hours on a typical weekday, Saturday, and Sunday (conditional on positive values) were 2.8 (±3.4), 3.0 (±3.7), and 3.1 (±3.8), respectively.

### Regression analysis

Results of linear FE regressions are shown in Table 2. As outcome variables, neuroticism (first column), extraversion (second column), openness to experience (third column), agreeableness (fourth column), and conscientiousness (fifth column) were used. All indicator variables were associated with an increase in neuroticism scores (except for 4 hours of informal care on a typical Saturday: β = .46, p < .10). As regards conscientiousness, only ‘5 hours and more’ on a typical Sunday was associated with an increase in conscientiousness (β = .32, p < .05). For the sake of readability, the control variables were not displayed in Table 2. On a typical weekday, for example, increasing age was associated with a decrease in all personality factors (neuroticism: β = -.08, p < .001; extraversion: β = -.03, p < .001; openness to experience: β = -.02, p < .001; agreeableness: β = -.04, p < .001; conscientiousness: β = -.04, p < .001). Moreover, while worsening self-rated health (e.g., changes from ‘very good’ to ‘good) was associated with an increase in neuroticism score (β = .54, p < .001), it was associated with a decrease in the other personality factors (extraversion: β = -.30, p < .001; openness to experience: β = -.22, p < .01; agreeableness: β = -.21, p < .01; conscientiousness: β = -.19, p < .01).

**Table 2:** Results of linear FE regressions (Wave 2005, wave 2009, and wave 2013) (with informal care hours as indicator variables).

Independent variables	Neuroticism (higher values indicate higher neuroticism)	Extraversion (higher values indicate higher extraversion)	Openness to experience (higher values indicate higher openness)	Agreeableness (higher values indicate higher agreeableness)	Conscientiousness (higher values indicate higher conscientiousness)	Neuroticism (higher values indicate higher neuroticism)	Extraversion (higher values indicate higher extraversion)	Openness to experience (higher values indicate higher openness)	Agreeableness (higher values indicate higher agreeableness)	Conscientiousness (higher values indicate higher conscientiousness)	Neuroticism (higher values indicate higher neuroticism)	Neuroticism (higher values indicate higher neuroticism)	Extraversion (higher values indicate higher extraversion)	Openness to experience (higher values indicate higher openness)	Agreeableness (higher values indicate higher agreeableness)
1 hour of informal caregiving on a typical weekday (Ref.: 0 hours)	0.43***	0.14	0.13	-0.11	-0.05										
	(0.10)	(0.09)	(0.10)	(0.09)	(0.08)										
2 hours of informal caregiving on a typical weekday	0.58***	0.08	0.02	-0.21	-0.04										
	(0.15)	(0.13)	(0.14)	(0.13)	(0.11)										
3 hours of informal caregiving on a typical weekday	0.59**	-0.15	-0.24	0.05	0.05										
	(0.22)	(0.18)	(0.22)	(0.18)	(0.19)										
4 hours of informal caregiving on a typical weekday	0.54*	0.07	0.19	-0.27	0.21										
	(0.26)	(0.26)	(0.29)	(0.27)	(0.22)										
≥ 5 hours of informal caregiving on a typical weekday	0.67**	-0.05	0.15	-0.07	0.27										
	(0.24)	(0.18)	(0.22)	(0.19)	(0.16)										
1 hour of informal caregiving on a typical Saturday (Ref.: 0 hours)						0.34***	0.11	0.13	-0.07	-0.13					
						(0.10)	(0.09)	(0.10)	(0.09)	(0.09)					
2 hours of informal caregiving on a typical Saturday						0.49***	0.18	0.13	-0.18	0.04					
						(0.14)	(0.12)	(0.13)	(0.11)	(0.10)					
3 hours of informal caregiving on a typical Saturday						0.58**	-0.09	-0.12	0.10	-0.01					
						(0.21)	(0.18)	(0.22)	(0.18)	(0.18)					
4 hours of informal caregiving on a typical Saturday						0.46+	0.07	0.08	-0.13	0.18					
						(0.27)	(0.24)	(0.25)	(0.23)	(0.20)					
≥ 5 hours of informal caregiving on a typical Saturday						0.69**	0.04	0.17	-0.02	0.28+					
						(0.23)	(0.17)	(0.20)	(0.18)	(0.15)					
1 hour of informal caregiving on a typical Sunday (Ref.: 0 hours)											0.39***	0.19*	0.25*	-0.05	-0.02
											(0.11)	(0.09)	(0.11)	(0.10)	(0.09)
2 hours of informal caregiving on a typical Sunday											0.40**	-0.06	-0.03	-0.27*	0.09
											(0.15)	(0.12)	(0.14)	(0.12)	(0.11)
3 hours of informal caregiving on a typical Sunday											0.56*	-0.01	-0.05	-0.02	-0.07
											(0.23)	(0.18)	(0.22)	(0.19)	(0.19)
4 hours of informal caregiving on a typical Sunday											0.62*	0.24	0.28	-0.12	0.42+
											(0.28)	(0.23)	(0.25)	(0.22)	(0.22)
≥ 5 hours of informal caregiving on a typical Sunday											0.52*	0.05	0.09	-0.01	0.32*
											(0.23)	(0.17)	(0.20)	(0.18)	(0.15)
Control variables Constant	✓	✓	✓	✓	✓	✓	✓	✓	✓	✓	✓	✓	✓	✓	✓
	15.58***	16.01***	14.92***	19.14***	18.21***	15.65***	16.04***	14.90***	19.14***	18.23***	15.59***	16.05***	14.92***	19.13***	18.26***
	(0.46)	(0.41)	(0.44)	(0.38)	(0.39)	(0.46)	(0.41)	(0.45)	(0.39)	(0.39)	(0.46)	(0.41)	(0.45)	(0.39)	(0.39)
Observations	55,047	55,041	54,741	55,043	54,868	54,704	54,701	54,405	54,700	54,530	54,695	54,689	54,395	54,691	54,520
Number of Individuals	31,714	31,713	31,592	31,716	31,625	31,619	31,620	31,498	31,622	31,535	31,623	31,622	31,503	31,625	31,539
R^2^	0.0316	0.00648	0.00462	0.00869	0.0117	0.0317	0.00657	0.00468	0.00856	0.0116	0.0313	0.00666	0.00475	0.00857	0.0117

Beta coefficients were reported; Cluster-robust standard errors in parentheses;

*** p<0.001,

** p<0.01,

* p<0.05,

^+^ p<0.10.

✓: All models are adjusted for age, marital status, educational level, employment status, income, self-rated health and disability.

Furthermore, in additional analysis we included period effects and squared age. In terms of significance and effect size, findings remained almost the same.

Lastly, in another specification a dummy variable indicating a positive amount of informal care hours (or not) and the informal caregiving hours (measured continuously) replaced our informal caregiving time categories (S1 Table). This dummy variable suggested that changes to informal care were associated with an increase in neuroticism. Compared with Table 2, there was an additional association between informal caregiving hours on a typical Saturday and conscientiousness (β = .04, p < .05).

## Discussion

Adjusting for potential confounders, FE regressions showed that whether or not someone provides informal care is markedly associated with changes in neuroticism. Given that an individual provides informal care, the actual number of care hours did not matter in most cases. Informal caregiving was not associated with openness to experience, extraversion and agreeableness. As regards conscientiousness, only ‘5 hours and more’ on a typical Sunday was associated with an increase in conscientiousness (β = .32, p < .05). Informal caregiving on a typical weekday or Saturday was not associated with changes in conscientiousness in our main model.

Initially, it was hypothesized that increases in informal caregiving time are associated with an increase in neuroticism, and conscientiousness, and a decrease in extraversion. However, regression analysis only confirmed the hypothesis of a longitudinal association between informal caregiving time and neuroticism. This appears plausible because it has been shown that starting informal care is associated with an increase in depressive symptoms [5] and an increase in neuroticism is associated with an increase in negative emotions and depressive symptoms.

The longitudinal association between an increase in informal caregiving time (‘5 hours and more’) and conscientiousness was only evident when informal caregiving hours on a typical *Sunday* was used as explanatory variable. This might be explained by the fact that a considerable increase in informal caregiving hours on a typical Sunday might indicate particularly that individuals become well-structured in order to manage various life domains on this day of rest (e.g., family obligations or housework). However, future research needs to clarify why a marked increase in informal caregiving hours on a typical weekday or Saturday is not associated with conscientiousness because informal caregivers usually have to combine (full-time) work and private care. The hypothesis of a longitudinal association between informal caregiving time and extraversion has to be rejected. It might be the case that while an increase in caregiving time leads to withdrawal or disinterest in some groups, it leads to an increased desire to escape caregiving tasks in other groups. Further research is required to test this hypothesis.

As one of few studies, based on a nationally representative sample, the present study investigated whether informal care was associated with personality factors using a longitudinal approach, adjusting for various potential confounders. Using FE regressions, the problem of unobserved heterogeneity—which is a fundamental problem particularly in cross-sectional studies—has been mitigated. However, FE regressions might be biased by unobserved time-varying factors. The present study was not restricted to compare caregivers with non-caregivers which might be biased by self-selection. Changes within informal caregiving time were used in the FE framework. Individuals were followed over a long period (2005 to 2013). The BFI-S demonstrated acceptable psychometric properties [26]. However, it has flaws for agreeableness.

For reasons of data availability, the present study was restricted to informal caregiving hours. Information is missing with regard to the dimensions of informal care (e.g., help around house, supervision or nursing care services). In addition, we cannot rule out that an increase in caregiving time decreases mental health, which in turn is associated with an increase in neuroticism. However, mental health was only assessed in even years from 2002 onwards. Therefore, we refrained from using this information in this study. Moreover, information is missing about the morbidity of care recipients. For example, an increase in caregiving time might reflect a decline in health of care recipients (e.g., cognitive decline [36]). Thus, we cannot rule out that our results reflect emotional effects of seeing the health decline of care-recipients (e.g., mother/father or spouse). Consequently, our findings should be interpreted with great caution. Notwithstanding, future research needs to account more explicitly for the dimensions of informal care and information about the care-recipient is required. In addition, the possibility of reverse causation (changes of personality factors cause changes in informal caregiving hours) cannot be excluded.

Given the assumptions that neither adaptation nor anticipation occurs, linear FE regressions correctly estimate the change in personality factors (under the assumption of strict exogeneity). However, it has been shown that anticipation and adaptation effects exist in various research areas disciplines [33–35]. These effects can distort results of regression analysis. In this study, anticipation and adaptation effects were not examined due to constraints in waves. Future studies are needed to clarify anticipation and adaptation effects in the longitudinal association between informal caregiving and personality factors.

In conclusion, our findings stress the longitudinal association between informal caregiving and neuroticism. Further research is necessary (e.g., based on instrumental variable approaches) to clarify the causal relationship of these factors.

There is also evidence that personality factors can moderate the relationship between informal caregiving and well-being outcomes [37, 38]. Consequently, future research is required to clarify the relationship between informal caregiving and personality in more detail.

## Supporting information

S1 Table Results of linear FE regressions (Wave 2005, wave 2009, and wave 2013) (with additional dummy variable for informal caregiving). (DOCX)
