# Supplementary material for: Informal caregiving and personality: Results of a population-based longitudinal study in Germany
Source: PLoS One. 2018 Sep 6;13(9):e0203586. doi: 10.1371/journal.pone.0203586 (PMC6126863; doi:10.1371/journal.pone.0203586)
Supplement: S1 Table — (DOCX) [file pone.0203586.s001.docx]

S1 Table. Results of linear FE regressions (Wave 2005, wave 2009, and wave 2013) (with dummy variable for informal caregiving and informal caregiving hours)

|  | (1) | (2) | (3) | (4) | (5) | (6) | (7) | (8) | (9) | (10) | (11) | (12) | (13) | (14) | (15) |
| --- | --- | --- | --- | --- | --- | --- | --- | --- | --- | --- | --- | --- | --- | --- | --- |
| Independent variables | Neuroticism (higher values indicate higher neuroticism) | Extraversion (higher values indicate higher extraversion) | Openness to experience (higher values indicate higher openness) | Agreeableness (higher values indicate higher agreeableness) | Conscientiousness (higher values indicate higher conscientiousness) | Neuroticism (higher values indicate higher neuroticism) | Extraversion (higher values indicate higher extraversion) | Openness to experience (higher values indicate higher openness) | Agreeableness (higher values indicate higher agreeableness) | Conscientiousness (higher values indicate higher conscientiousness) | Neuroticism (higher values indicate higher neuroticism) | Extraversion (higher values indicate higher extraversion) | Openness to experience (higher values indicate higher openness) | Agreeableness (higher values indicate higher agreeableness) | Conscientiousness (higher values indicate higher conscientiousness) |
|  |  |  |  |  |  |  |  |  |  |  |  |  |  |  |  |
| Positive amount of informal care hours on a typical weekday (Ref.: Not providing informal care on a typical weekday) | 0.45*** | 0.07 | 0.13 | -0.11 | -0.07 |  |  |  |  |  |  |  |  |  |  |
|  | (0.10) | (0.08) | (0.10) | (0.08) | (0.08) |  |  |  |  |  |  |  |  |  |  |
| Informal care (hours on a typical weekday) | 0.02 | 0.00 | -0.02 | -0.00 | 0.03+ |  |  |  |  |  |  |  |  |  |  |
|  | (0.03) | (0.02) | (0.03) | (0.02) | (0.02) |  |  |  |  |  |  |  |  |  |  |
| Positive amount of informal care hours on a typical Saturday (Ref.: Not providing informal care on a typical Saturday) |  |  |  |  |  | 0.40*** | 0.07 | 0.16 | -0.07 | -0.11 |  |  |  |  |  |
|  |  |  |  |  |  | (0.10) | (0.08) | (0.10) | (0.08) | (0.08) |  |  |  |  |  |
| Informal care (hours on a typical Saturday) |  |  |  |  |  | 0.02 | 0.01 | -0.02 | -0.00 | 0.04* |  |  |  |  |  |
|  |  |  |  |  |  | (0.03) | (0.02) | (0.02) | (0.02) | (0.02) |  |  |  |  |  |
| Positive amount of informal care hours on a typical Sunday (Ref.: Not providing informal care on a typical Sunday) |  |  |  |  |  |  |  |  |  |  | 0.42*** | 0.07 | 0.18+ | -0.11 | -0.03 |
|  |  |  |  |  |  |  |  |  |  |  | (0.10) | (0.09) | (0.10) | (0.09) | (0.08) |
| Informal care (hours on a typical Saturday) |  |  |  |  |  |  |  |  |  |  | 0.01 | 0.01 | -0.02 | 0.01 | 0.04* |
|  |  |  |  |  |  |  |  |  |  |  | (0.03) | (0.02) | (0.02) | (0.02) | (0.02) |
|  |  |  |  |  |  |  |  |  |  |  |  |  |  |  |  |
| Control variables | 🗸 | 🗸 | 🗸 | 🗸 | 🗸 | 🗸 | 🗸 | 🗸 | 🗸 | 🗸 | 🗸 | 🗸 | 🗸 | 🗸 | 🗸 |
|  |  |  |  |  |  |  |  |  |  |  |  |  |  |  |  |
| Constant | 15.10*** | 16.10*** | 15.04*** | 19.15*** | 18.33*** | 15.16*** | 16.13*** | 15.03*** | 19.16*** | 18.35*** | 15.10*** | 16.14*** | 15.04*** | 19.15*** | 18.38*** |
|  | (0.46) | (0.41) | (0.44) | (0.38) | (0.39) | (0.46) | (0.41) | (0.45) | (0.39) | (0.39) | (0.46) | (0.41) | (0.45) | (0.39) | (0.39) |
|  |  |  |  |  |  |  |  |  |  |  |  |  |  |  |  |
| Observations | 55,047 | 55,041 | 54,741 | 55,043 | 54,868 | 54,704 | 54,701 | 54,405 | 54,700 | 54,530 | 54,695 | 54,689 | 54,395 | 54,691 | 54,520 |
| Number of Individuals | 31,714 | 31,713 | 31,592 | 31,716 | 31,625 | 31,619 | 31,620 | 31,498 | 31,622 | 31,535 | 31,623 | 31,622 | 31,503 | 31,625 | 31,539 |
| R² | 0.0313 | 0.00591 | 0.00434 | 0.00824 | 0.0116 | 0.0313 | 0.00600 | 0.00448 | 0.00814 | 0.0115 | 0.0309 | 0.00602 | 0.00444 | 0.00811 | 0.0116 |

Beta coefficients were reported; Cluster-robust standard errors in parentheses; *** p<0.001, ** p<0.01, * p<0.05, + p<0.10. All models are adjusted for age, marital status, educational level, employment status, income, self-rated health and disability.
